# Targeting the Mitochondrial Metabolic Network: A Promising Strategy in Cancer Treatment

**DOI:** 10.3390/ijms21176014

**Published:** 2020-08-21

**Authors:** Luca Frattaruolo, Matteo Brindisi, Rosita Curcio, Federica Marra, Vincenza Dolce, Anna Rita Cappello

**Affiliations:** Department of Pharmacy, Health and Nutritional Sciences, University of Calabria, Via P. Bucci, 87036 Rende (CS), Italy; luca.frattaruolo@unical.it (L.F.); matteo.brindisi@unical.it (M.B.); rosita.curcio@unical.it (R.C.); federica.marra@unical.it (F.M.); vincenza.dolce@unical.it (V.D.)

**Keywords:** mitochondria, metabolic rewiring, metabolic network, targeting mitochondria, cancer therapy

## Abstract

Metabolic reprogramming is a hallmark of cancer, which implements a profound metabolic rewiring in order to support a high proliferation rate and to ensure cell survival in its complex microenvironment. Although initial studies considered glycolysis as a crucial metabolic pathway in tumor metabolism reprogramming (i.e., the Warburg effect), recently, the critical role of mitochondria in oncogenesis, tumor progression, and neoplastic dissemination has emerged. In this report, we examined the main mitochondrial metabolic pathways that are altered in cancer, which play key roles in the different stages of tumor progression. Furthermore, we reviewed the function of important molecules inhibiting the main mitochondrial metabolic processes, which have been proven to be promising anticancer candidates in recent years. In particular, inhibitors of oxidative phosphorylation (OXPHOS), heme flux, the tricarboxylic acid cycle (TCA), glutaminolysis, mitochondrial dynamics, and biogenesis are discussed. The examined mitochondrial metabolic network inhibitors have produced interesting results in both preclinical and clinical studies, advancing cancer research and emphasizing that mitochondrial targeting may represent an effective anticancer strategy.

## 1. Mitochondria and Cancer

For many years, glycolysis has been considered the engine of cancer cells, the metabolic pathway that ensures their rapid production of ATP and supports an uncontrolled proliferation. Indeed, the Warburg studies showed that cancer metabolic reprogramming shifts energy metabolism towards the glycolytic pathway even in aerobic conditions [1]. Such aerobic glycolysis conditions, defined as the Warburg effect, ensures a rapid production of ATP from glucose, albeit less efficiently than complete mitochondrial oxidation, a process coupled with oxidative phosphorylation. The maintenance of the glycolytic pathway in cancer is associated with the conversion of the high amount of produced pyruvate into lactate, in order to guarantee a high NAD^+^/NADH cell ratio, which is essential for feeding glycolysis. The clinical application of the Warburg effect has led to the development of imaging technologies allowing the identification of malignant neoplasms by evaluating the ability of cells to metabolize 2-[18F] fluoro-2-deoxy-d-glucose (18F-FDG), a glucose analogue easily traceable by positron emission tomography (PET).

Based on Warburg’s observations, for a long time, aerobic glycolysis of cancer cells was believed to be the result of a mitochondrial dysfunction, which in turn was considered to be a cause of cancer. Numerous nuclear–cytoplasmic transfer experiments supported this theory, showing that normal cytoplasm suppresses tumorigenesis in cell cybrids [2]. In particular, several in vitro and in vivo studies showed that the fusion of healthy enucleated cells (cytoplasts) with tumoral karyoplastes can significantly reduce tumorogenicity with respect to parental cancer. In contrast, the fusion of tumor cytoplasts with healthy karyoplasts does not determine any reduction of tumorogenicity, suggesting that tumor development is triggered by mitochondrial rather than nuclear dysfunctions.

**Figure 1:**
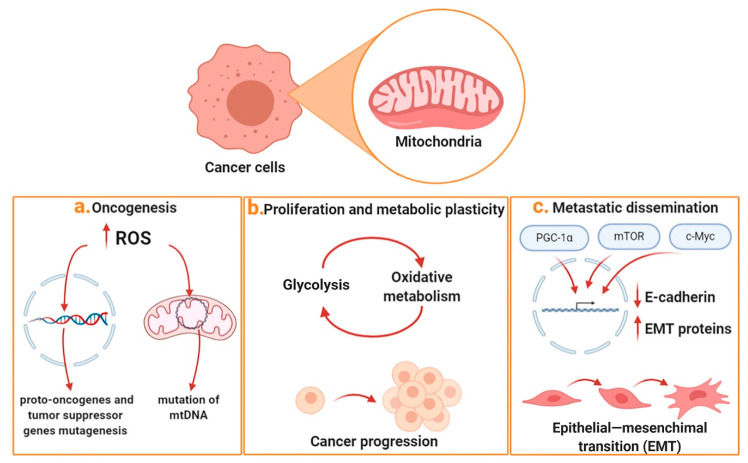
Mitochondrial relevance to cancer progression. Mitochondria play an important role in the main processes that characterize tumor progression. The figure shows the main effects of mitochondria in the three main steps characterizing cancer development: (**a**) ROS production is capable of causing damage at the levels of nuclear and mitochondrial DNA, thus promoting oncogenesis; (**b**) metabolic plasticity is responsible for greater tumor progression and stemness; (**c**) biogenesis and high mitochondrial turnover are responsible for EMT and metastatic dissemination.

In recent decades, however, the Warburg effect has been overcome as the dogma of cancer biochemistry, with hypotheses emerging that the mechanisms underlying tumor metabolic reprogramming are much more complex than those theorized by Warburg, and that mitochondrial metabolism can change during oncological pathology, thus influencing neoplastic transformation, tumor progression, and metastatic dissemination processes (Figure 1).

Mitochondrial metabolism plays a key initial role during oncogenesis. A lot of studies have highlighted that the mitochondrial generation of reactive oxygen species (ROS), subproducts of respiration, favors DNA damage. This can result in mutagenesis able to affect proto-oncogenes and tumor-suppressor genes, leading to self-feeding tumor proliferation [3]. In this regard, in many tumors, an increase in ROS production has been related to mutations found in the mitochondrial DNA (mtDNA), encoding for several subunits constituting the electron transport chain complexes [4]. Over the years, in addition to ROS, other metabolites, intermediates of mitochondrial metabolism, have been believed to trigger oncogenesis and neoplastic transformation processes, and these molecules have been defined as oncometabolites [5,6,7]. In particular, an accumulation of succinate, fumarate, and 2-hydroxy-glutarate (2-HG) has been found in several tumors. The accumulation of succinate and fumarate is mainly driven by inactivating mutations concerning the succinate dehydrogenase (SDH) and fumarate hydratase (FH) enzymes, respectively. These mutated enzymes, in their inactive forms, do not allow the normal progression of TCA cycle which, in turn, induces the accumulation of their specific substrates. On the other hand, the accumulation of 2-HG is due to mutations found in the isocitrate dehydrogenase isoforms 1 and 2 (IDH1 and IDH2), which are normally responsible for converting isocitrate into α-keto-glutarate (α-KG). When IDH1 and IDH2 are mutated, a strong accumulation of 2-HG is observed in the presence of NADPH. These oncometabolites share the ability to inhibit (α-KG)-dependent enzymes that mediate gene expression at the epigenetic level, and the triggering of pseudo-hypoxia conditions mediated by hypoxia inducible factor 1 (HIF1).

The second level at which mitochondrial metabolism plays a fundamental role is in tumor progression. The uncontrolled proliferation of cancer cells makes the supply of anabolic substrates for the synthesis of DNA, proteins, and lipids necessary for the cell. In this regard, the mitochondrial cycle of tricarboxylic acids (TCA) plays a key role, since it provides fundamental intermediates for anabolic processes. Among these, a key intermediate is citrate, which exits the mitochondria and supplies the cytosolic environment with the acetyl-CoA necessary for fatty-acid and cholesterol biosynthesis, as well as for gene expression regulation at the epigenetic level (histone acetylation). In this regard, the importance of the mitochondrial citrate carrier (SLC25A1) has recently emerged in tumor progression, cancer stemness, and resistance to therapy; additionally, the specific SLC25A1 inhibitor benzenetricarboxylate (BTA) has been proven to exert a promising anticancer activity [8,9,10].

The fermentation of pyruvate to lactate, which characterizes the Warburg effect, contributes to depletion of the TCA cycle of anabolic substrates, even if in cancer, a strengthening of the anaplerotic pathways responsible for their regeneration occurs. Indeed, in many tumors, a potentiation of glutaminolysis has been observed. Although the glutamate generated by the deamination of glutamine represents by itself an anabolic intermediate, its mitochondrial conversion into α-keto-glutarate is fundamental to supplying the TCA cycle of anabolic intermediates such as succinyl-CoA, oxaloacetate and citrate [11,12,13]. Moreover, it is noteworthy that in several tumors, the activation of glutaminolysis is responsible for the establishment of a truncated TCA cycle. In these, one of the five carbon atoms of the α-keto-glutarate, derived from glutamate, is removed as CO_2_, while the remaining four leave the mitochondria in the form of malate, which can give rise to several metabolic pathways in the cytosol.

During the progression of the neoplastic pathology, a tumor acquires a high metabolic plasticity due to its interaction with the microenvironment, characterized by the presence of nonmalignant cells. Metabolic plasticity is therefore conferred by the sum of the oncogenic effects intrinsic to the tumor and the metabolic effects induced by the surrounding cells. In this regard, many studies have highlighted how fibroblasts and other cell types present in the microenvironment are stimulated to produce and release metabolites used by tumor cells as sources of carbon atoms, such as fatty acids, lactate, and alanine, which support tumor progression [14,15,16]. The relationship established between the tumor and microenvironment can therefore be defined as parasitic. In contrast, within the tumor itself, a symbiotic relationship is established among the different cells composing the cancer, and this symbiosis is responsible for cancer metabolic heterogeneity. The less hypoxic cells shift their metabolism towards oxidative phosphorylation, to fulfil the high energy needs. To this end, they employ the carbon sources secreted by the microenvironment without competing for the use of glucose with the most hypoxic cells, for which glycolysis remains the only available energy pathway.

Another level that strongly involves mitochondrial metabolism is the metastatic dissemination process. Although macro-metastases can be considered glycolytic entities, since they are clinically detected through the use of 18F-FDG, mitochondrial metabolism plays a key role in the metastasis process [17]. In fact, the first step in the metastasis process is the epithelial–mesenchymal transition (EMT), which is strongly promoted by mitochondrial biogenesis and by oxidative phosphorylation (OXPHOS) [18,19,20]. In this regard, several studies have shown that Peroxisome proliferator-activated receptor gamma coactivator 1-alpha (PGC-1α), mammalian target of rapamycin (mTOR), and c-Myc pathways regulate biogenesis and mitochondrial activity in metastatic cells. These pathways are able to suppress the expression of E-cadherin, to stimulate the expression of epithelial–mesenchymal-transition-associated factors, such as vimentin and Snail, and also to positively regulate the Transforming Growth Factor-β1 (TGF-β1)-Induced EMT [21,22,23,24].

Based on the role that mitochondria play in neoplastic progression, it is clear how molecules capable of interacting with mitochondrial targets can exert an antitumor activity. The mitochondrial metabolic network, however, is very complex and subjected to intense rewiring in cancer. Over the years, more and more knowledge has been acquired on tumor metabolic reprogramming, and new potential targets have been identified as well as new small molecules capable of acting on them. In this review, the mitochondrial targets of the main metabolic pathways, of which pharmacological inhibition has proven to be promising in preclinical and clinical oncological studies, are discussed.

## 2. Targeting Mitochondrial Metabolism in Cancer

### 2.1. Targeting OXPHOS

In recent years, many experimental reports have highlighted that several types of cancer cells fuel tumorigenesis by using OXPHOS. In this light, such a metabolic pathway represents one of the most studied mitochondrial targets in the search for new potential anticancer candidates. OXPHOS activity is differently modulated depending on tumor type. OXPHOS downregulation has been observed in several cancer types, due to a reduced mtDNA content or to mutations found in it; at the same time, the glycolytic pathway appeared to be upregulated [25]. On the other hand, several studies have highlighted that OXPHOS can be upregulated in different tumors, such as lymphomas, leukemias, melanomas, and breast, pancreatic, lung, and endometrial cancers. Such OXPHOS upregulation was found to be in agreement with the above-mentioned studies, which highlighted an increased mitochondrial activity related to tumorigenesis, as well as to anchorage-independent growth and resistance to chemotherapy [25]. Remarkably, the amount of mtDNA or the expression of OXPHOS genes may not necessarily reflect the rate of OXPHOS function; hence, it is necessary to evaluate OXPHOS activity by using different approaches. In this regard, despite the decrease in mtDNA content that was observed in breast cancer [25], Guppy et al. measured the amount of ATP generated by glycolysis and OXPHOS in MCF-7 breast cancer cells, highlighting that 80% of ATP comes from OXPHOS and 20% from glycolysis. Moreover, a study performed in human patients in order to analyze metabolic pathways’ flow in non-small-cell lung cancer (NSCLC) displayed that high rates of both glycolysis and oxidative metabolism can be observed in this cancer type, highlighting a metabolic heterogeneity strongly influenced by microenvironment. In addition, Sohoni et al. highlighted that the elevated OXPHOS activity and tumorigenicity characterizing NSCLC cells are related to elevated both flux and function of heme, a key molecule in mitochondrial OXPHOS [26].

**Figure 2:**
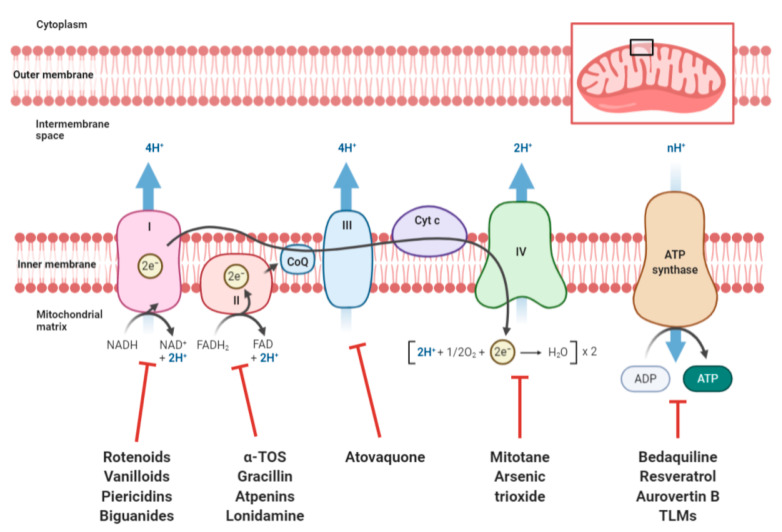
OXPHOS as promising target to affect mitochondrial activity in cancer cells. ETC complexes, exploiting the oxidation of NADH and FADH_2_ from TCA and glycolysis, generate an electrochemical gradient able to determine ATP production through F_1_F_O_-ATPase. In the figure: the different components of the ETC, the F1Fo-ATPase, and the molecules discussed in this review, which have exhibited promising results in vitro and in vivo in inhibiting their respective targets, are depicted in detail.

The pharmacological impairment of the functionality of one of the electron transport chain complexes or of the F_1_F_O_-ATPase results not only in a reduction in the cellular energy supply, but also in a loss of the mitochondrial membrane potential generated by the complexes I, III, and IV, with a consequent alteration of the mitochondrial permeability followed by the release of pro-apoptotic factors triggering programmed cell death [27,28,29]. The reduction in the flow of electrons through the various complexes of the respiratory chain is also responsible for increased production of ROS, and in particular of the superoxide anion (O_2_^−^), a subproduct of mitochondrial respiration generated by the incomplete reduction of O_2_ to H_2_O. Superoxide anions, together with the secondary ROS (OH and H_2_O_2_), may interact with mitochondrial macromolecules such as membranes, proteins, and mtDNA, further impairing mitochondrial function and promoting pro-apoptotic processes. Many naturally occurring molecules and synthetic compounds are able to inhibit oxidative phosphorylation at different levels (Figure 2). Many of these are FDA-approved drugs currently used in nontumor therapies, for which repurposing is being evaluated in the oncological field.

NADH–ubiquinone oxidoreductase (complex I) inhibitors are very heterogeneous from the chemical point of view. These inhibitors can contain hydroquinone/quinone motifs [30], such as rotenoids, vanilloids, and piericidins, but they can also occur as very different molecules, such as metformin and other biguanides, capable of binding to different domains of this enzymatic complex, determining a noncompetitive inhibition [31,32]. This latter class of compounds has aroused particular interest in recent years, proving to be a promising scaffold for anticancer candidates, active at sub-nanomolar concentrations. Regardless of their chemical class, complex I inhibitors, alone or in association with molecules shifting tumor metabolism towards oxidative phosphorylation, have shown a high antitumor potential against different types of cancer cells studied both in in vitro and in vivo models [33,34,35,36].

The respiratory chain complex II, known as succinate dehydrogenase (SDH), represents the connection point between the Krebs cycle (TCA) and the electron transport chain (ETC). Unlike the other complexes, succinate dehydrogenase is not involved in the transfer of protons from the matrix to the intermembrane space, and therefore does not directly contribute to the creation of the mitochondrial membrane potential. However, recent studies have highlighted the connection of this complex with the apoptotic pathway mediated by death receptors [37,38]. Complex II inhibitors, such as α-tocopheryl succinate (α-TOS) [39], gracillin [40], and atpenins [41,42,43], were found to increase ROS production, triggering apoptotic death in vitro and in vivo. Furthermore, in recent studies, lonidamine, an FDA-approved drug initially classified as a hexokinase inhibitor, was shown to strongly inhibit SDH. Although it is endowed with a poor anticancer activity by itself, lonidamine is able to enhance the activity of chemotherapeutic agents currently used in clinical practice, such as DNA-alkylating agents [44,45] and anthracyclines [46].

Among the molecules capable of inhibiting complex III, known as cytochrome c reductase, there is another FDA-approved drug, atovaquone, which is used in clinical practice for the treatment of malaria and pneumocystis pneumonia infections. Given its structural analogy to coenzyme Q10, atovaquone is able to competitively inhibit complex III, reducing tumor proliferation in vitro and in vivo [47,48] and inhibiting the propagation of cancer stem cells [49], a subpopulation of cancer cells highly dependent on mitochondrial metabolism and largely responsible for tumor recurrence and metastasis.

The inhibition of complex IV of the electron transport chain, cytochrome c oxidase, has proven to be an effective therapeutic strategy in the treatment of some tumors to date. In this regard, mitotane is currently the only treatment available for adrenocortical cancer (ACC) [50], and recent studies have shown that its antitumor activity arises from OXPHOS impairment due to the inhibition of the complex IV [51]. This complex is also pharmacologically inhibited by arsenic trioxide, an FDA-approved drug used for acute promyelocytic leukemia treatment and currently being studied in other tumors, in which it has been found to reduce cancer stemness and to improve radiation therapy response [52,53,54,55].

To date, the role of F_1_F_O_-ATPase in cancer is controversial [56]. This enzymatic complex is actively involved in ATP production at the mitochondrial level, and in many tumors its expression appears to be reduced [57]. In addition, a high expression of IF1 peptide, an endogenous inhibitor of ATP synthase, has been found in several tumors, appearing to be a negative prognostic factor. On the other hand, in other tumors such as breast cancer, an opposite trend has been observed, and IF1 has been shown to be a positive prognostic factor, which appears to be strongly downregulated in triple-negative breast cancer cells [58].

F_1_F_O_-ATPase, however, seems to have a very important role in cancer. In addition to ensuring the synthesis of ATP by exploiting the proton gradient generated by the ETC, it is able to reverse its catalytic activity in conditions of severe hypoxia. ATP hydrolysis catalyzed by the reversion of ATP synthase can guarantee, even in the absence of ETC functionality, an optimal mitochondrial membrane potential, which is necessary for the survival of cancer cells which otherwise would undergo apoptotic death mechanisms [59,60].

Several F_1_F_O_-ATPase inhibitors have recently proven to be potential anticancer candidates. Bedaquiline, an FDA-approved antibiotic used for the treatment of multidrug-resistant pulmonary tuberculosis, has been shown to reduce OXPHOS and glycolytic metabolism in tumor cells by inhibiting ATP synthase, as well as suppressing the proliferative expansion of cancer stem cells [61] and tumor angiogenesis [62].

Various natural products have also been shown to have an inhibitory activity against F_1_F_O_-ATPase, exerting a significant antiproliferative/cytotoxic effect. These compounds include resveratrol and related polyphenols [63], aurovertin B [64], and thioviridamide-like molecules (TLMs), which have been shown to alter metabolism in cancer cells, to induce oxidative stress, and to inhibit tumor proliferation in vitro and in preliminary in vivo studies [65,66,67].

Furthermore, the inhibition of OXPHOS appears to be a promising therapeutic strategy for eradicating oncogene-addicted cancers, the treatment of which involves specific targeted therapies using tyrosine kinase inhibitors. However, onset of resistance often underlies the failure of such therapeutic strategies. Inhibitors of oncogene products, such as the epidermal growth factor receptor (EGFR), the anaplastic lymphoma kinase (ALK), the ABL tyrosine kinase, BRAF, and JAK2 kinases, are able to inhibit glycolysis; consequently, drug-resistant oncogene-addicted cancer cells exhibit higher aerobic mitochondrial respiration, display higher stemness, and are metabolically reprogrammed to depend on OXPHOS for survival. Strict reliance on OXPHOS represents a metabolic vulnerability of such cancer cells. In this regard, they can be treated with OXPHOS inhibitors, which in turn can resensitize cancer cells with acquired resistance to the respective targeted therapy [68,69].

Several studies have shown that a greater feedback activation of signal transduction activator of transcription 3 (STAT3) is at the basis of the resistance mechanisms of oncogene-addicted cancers and their associated metabolic reprogramming. STAT3 can localize to mitochondria and interact with GRIM-19, thus positively regulating the assembly and function of ETC complexes and stimulating OXPHOS [70,71]. In this regard, targeting STAT3 with small molecules acting at different levels of this pathway, such as OPB-51602, reduces OXPHOS activity and results in mitochondrial dysfunction, proving to be an effective strategy to overcome metabolic reprogramming characterizing resistant oncogene-addicted cancers [72,73,74].

However, a serious challenge in the clinical development of OXPHOS inhibitors is the potential toxicity that a nonspecific mitochondrial inhibition could trigger, such as peripheral neuropathy and lactic acidosis [75]. For this reason, the continuous identification of new compounds capable of directly or indirectly targeting OXPHOS while maintaining an acceptable toxicity profile is a main topic in cancer research.

### 2.2. Targeting Heme

**Figure 3:**
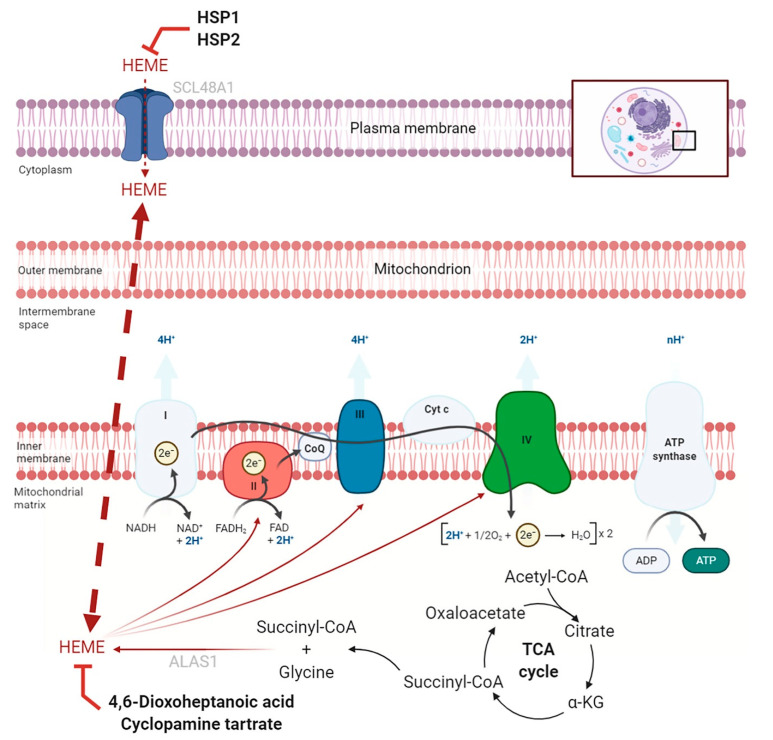
Targeting heme flux in cancer cells. The figure schematically shows heme uptake and biosynthesis, as well as its incorporotation into complexes II, III, and IV of the electron transport chain. The major heme flux inhibitors discussed in this review and their targets are shown.

Another important mitochondria-related pathway, of which the correlation with cancer has been increasingly studied in recent years, is the biosynthesis of heme, also called iron protoporphyrin IX. This important prosthetic group, responsible for the functionality of various heme proteins, can derive from the diet, uptaken by cells thanks to Heme carrier protein 1 (HCP1) and Heme transporter HRG1, or can be biosynthesized through a multienzymatic process straddling the cytosol and mitochondria, the limiting stage of which is represented by the activity of the enzyme 5-aminolevulic acid synthase (ALAS1) (Figure 3). This mitochondrial enzyme is responsible for the first biosynthetic reaction of heme, the condensation of glycine and succinyl-CoA coming from the TCA cycle [76]. Due to its ability to interact with oxygen, the heme group is present in several proteins with various functions, and plays a very important role in the oxidative phosphorylation process. Indeed, ETC complexes II, III, and IV possess heme groups. Another key protein in the oxidative phosphorylation process, related to the biosynthesis of heme, is Adenine nucleotide translocase (ANT2), which exchanges ATP for ADP across the inner mitochondrial membrane. It has, in fact, been shown that this protein is also responsible for the transport of heme precursors such as protoporphyrin IX within the mitochondrion, and its ADP/ATP transport activity is negatively regulated by the presence of heme or its precursors [77].

Several studies have correlated a high heme dietary intake through the consumption of red and processed meat with the pathogenesis of different carcinomas, such as esophageal, gastric, colorectal, breast, endometrial, pancreatic, and lung cancer. In addition, increased heme biosynthesis and uptake have been found in several tumors, including non-small-cell lung cancer, in which high heme levels, as well as an increased activity of heme proteins, have been shown to underlie metabolic rewiring and tumor progression [26,78,79]. This evidence highlights the important role in cancer of heme uptake, as well as of its biosynthesis and incorporation into oxygen-utilizing heme proteins, and has led to the study of molecules targeting these processes as a new anticancer strategy [80] (Figure 3).

In this regard, 4,6-dioxoheptanoic acid, also known as succinylacetone, proved to be a potent inhibitor of heme biosynthesis, with a potential anticancer activity. By inhibiting 6-aminolevulinic acid dehydratase, this compound induced heme depletion and mitochondrial dysfunction, thus suppressing respiration and proliferation in leukemia and cervical, colon, and lung cancer models [78,81,82,83,84]. The limited results obtained from in vivo studies, however, have led in recent years to attempts to reduce intracellular heme levels via different strategies. Cyclopamine tartrate, a known modulator of hedgehog signaling, was shown to suppress lung cancer oxygen consumption and proliferation, both in cultured cells and orthotopic tumor xenografts, by inhibiting heme metabolism and OXPHOS function [85]. Furthermore, a different strategy recently adopted is represented by the use of heme-sequestering peptides. By making appropriate modifications to the amino acid sequence of *Yersinia pestis* hemophore HasA, new heme-sequestering peptides, namely HSP1 and HSP2, have been developed and shown to reduce proliferation, migration, and invasion of non-small-cell lung cancer cells both in vitro and in vivo [26].

### 2.3. Targeting the TCA Cycle and Glutaminolysis

**Figure 4:**
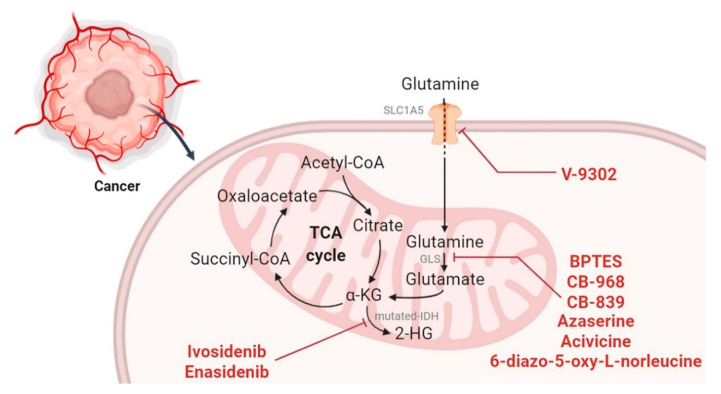
Targeting TCA and glutaminolysis in cancer cells. Glutaminolysis supplies the TCA cycle with anabolic intermediates to sustain a high proliferate rate in cancer cells. The figure shows the main stages of TCA and glutaminolysis inhibited by the molecules discussed in this review, which have displayed promising results in vitro and in vivo.

Although glycolysis plays a central role in tumor energy metabolism, in cancer cells, the tricarboxylic acid (TCA) cycle is kept active in order to generate anabolic substrates and oncometabolites, which together support neoplastic proliferation. Since the pyruvate produced by glycolysis is shifted towards lactic fermentation, instead of being converted into acetyl-CoA and entering the mitochondria, glutaminolysis supplies intermediates required for the TCA cycle. The connection between these two metabolic pathways is fundamental to the survival and proliferation of different tumors; therefore, it represents a possible target for cancer therapy (Figure 4).

One of the most studied therapeutic strategies is the inhibition of isocitrate dehydrogenase (IDH), which is mutated in several tumors such as gliomas and leukemia, and it is responsible for the production of the oncometabolite 2-HG [86,87,88]. Over the years, several molecules have been developed, able to selectively inhibit one of the different mutated IDH isoforms by reducing 2-HG levels and stimulating the differentiation of mutant IDH1/IDH2 acute myeloid leukemia (AML) cells [89,90,91,92]. Among the inhibitors of the IDH1 mutated isoform currently used in clinical settings, there is ivosidenib (AG-120), which was FDA-approved in 2018 for the treatment of relapsed or refractory AML with a susceptible IDH1 mutation. Furthermore, this drug was approved in 2019 as front-line therapy for newly diagnosed AML patients older than 75 years or with comorbidities precluding intensive chemotherapy. On the other hand, the mutated-IDH2 inhibitor enasidenib (AG-221) was approved by the FDA in 2017 for the treatment of relapsed or refractory AML specifically with an IDH2 mutation.

The glutaminolysis process, an anaplerotic pathway strongly activated in various tumors in order to supply the TCA cycle with intermediates, is mainly regulated by the transcription factor c-Myc [93,94]. This factor has been found to be upregulated in many types of cancers [95,96], and it is able to positively modulate glutamine uptake, as well as the expression of the enzymes responsible for its metabolism. There are several carriers able to transport this amino acid across the plasma membrane, while the presence of carriers that mediate its transit across the inner mitochondrial membrane remains controversial, and is still subject of study [97]. Many studies have shown that the plasma membrane carrier SLC1A5 is overexpressed in several tumors under the control of the c-Myc transcription factor, thus highlighting the important role played by this transporter in enhancing glutamine utilization in order to support cancers’ high proliferative rates [98,99,100,101,102]. Furthermore, a very recent study showed that a SLC1A5 carrier variant expressed under hypoxic conditions localizes to mitochondria and regulates the entry of glutamine, thus stimulating glutaminolysis and playing a key role in tumor metabolic reprogramming [103]. Further studies showed that the loss of function of the SLC1A5 carrier results in cell growth inhibition, autophagy, and apoptosis triggering in solid and hematopoietic malignancies [104,105]. Moreover, the pharmacological inhibition of this carrier, mediated by the compound V-9302, was responsible for a reduced tumor proliferation and an increased oxidative stress that triggers cell death in several in vitro and in vivo models [106]. 

In addition to inhibiting glutamine uptake, in the past decade, research has focused on the antitumor effects of inhibition of glutaminase (GLS), the enzyme responsible for hydrolytic deamidation of glutamine to glutamate in the first enzymatic reaction of glutaminolysis. There are many small molecules that in recent years have proven to be capable of inhibiting GLS [107,108], such as the naturally occurring molecules 6-diazo-5-oxy-L-norleucine, azaserine, and acivicin, and the synthetic compounds BPTES, 968 and CB-839 [109] (Figure 4). Among these, the compound CB-839 is a noncompetitive GLS inhibitor able to inhibit the entry of substrates into the TCA cycle, thus reducing cell viability and cancer stemness, increasing chemosensitivity, and inducing apoptotic cell death in several cancer types [110,111,112,113,114]. The promising antitumor potential of this compound makes it the only GLS inhibitor currently undergoing clinical studies (trial IDs: NCI-2018-00876, NCI2019-01365, NCI-2019-00572).

### 2.4. Targeting Mitochondrial Biogenesis

**Figure 5:**
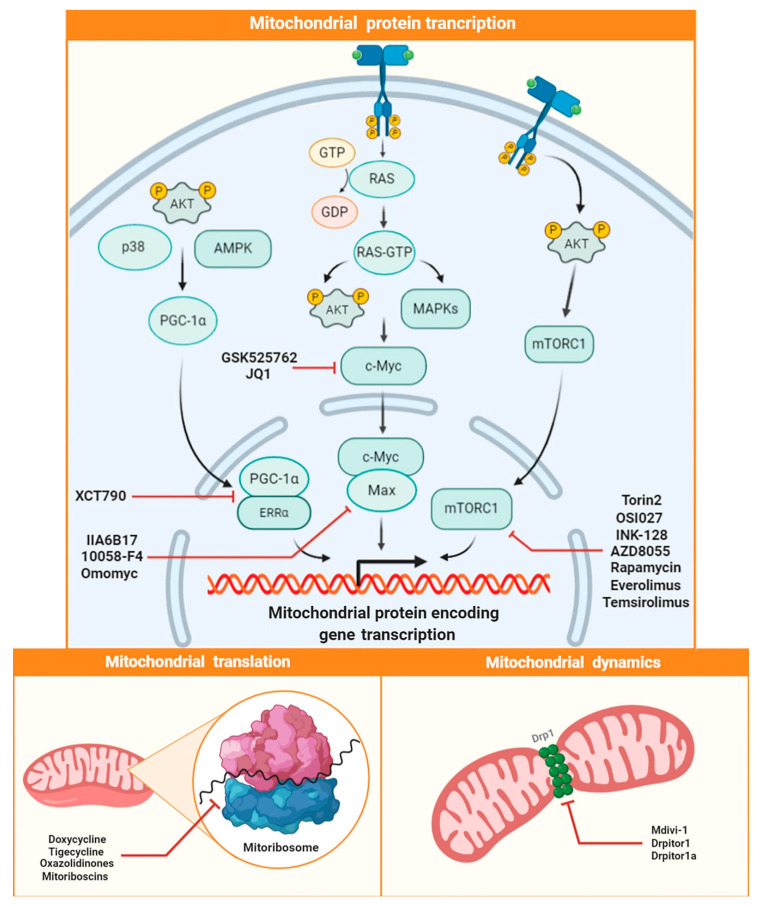
Targeting mitochondrial biogenesis. High mitochondrial efficiency, necessary to ensure a high proliferation and stemness, is guaranteed by high biogenesis, turnover, and mitochondrial dynamics. The figure shows the level at which the different molecules discussed in this review modulate mitochondrial protein expression (at transcriptional and translational level) as well as mitochondrial dynamics.

A possible target in controlling functions of the cancer powerhouse is represented by systems controlling mitochondrial biogenesis and turnover. Indeed, there are many pathways that, in a concerted way, may ensure an optimal level of mitochondrial efficiency in tumor cells [115,116]. In recent years, antitumor research has focused on the dense network enhancing biogenesis and mitochondrial efficiency, which are features associated with a poor prognosis in different types of cancer [117]. Mitochondrial biogenesis depends on the synthesis of different mitochondrial proteins, which is finely regulated at the levels of transcription and translation of both nuclear and mitochondrial genes (Figure 5).

Expression of mitochondrial proteins is a fundamental process supporting mitochondrial biogenesis, and it is transcriptionally regulated by PGC-1α and c-Myc. These proteins can act directly or indirectly (i.e., by interacting with other transcription factors), in order to regulate the expression of numerous genes that encode mitochondrial proteins. On this basis, such proteins are known to be of fundamental importance for the metabolic reprogramming that occurs in cancer cells. Several studies have shown that the increased activation of these pathways in cancer cells stimulates tumor proliferation [118] and survival in anchorage-independent conditions [119], which lies at the basis of neoplastic dissemination [120]. Another fundamental target in mitochondrial biogenesis regulation is represented by mTOR [121,122]. In more detail, mTOR is the catalytic subunit of mTOR complex 1 (mTORC1) and complex 2 (mTORC2), which are downstream components of the PI3K/AKT pathway, playing a central role in controlling cell proliferation and energy homeostasis [123,124].

All the previously mentioned signaling pathways are interconnected and work in concert with each other, thus forming a dense network able to ensure an adequate synthesis of mitochondrial proteins. Along with the mitochondrial fission–fusion processes, this guarantees a continuous turnover and high mitochondrial efficiency, which are essential for tumor proliferation. Based on these lines of evidence, several compounds have been developed over the years that are able to modulate mitochondrial biogenesis by destroying this important network, thus promoting a remarkable antitumor activity.

PGC-1α represents the central pivot of mitochondrial biogenesis, and it is a widely studied target by researchers engaged in cancer research [125]. This protein is a transcriptional coactivator that targets multiple transcription factors, such as nuclear respiratory factors (NRF-1 and NRF-2) and the estrogen-related receptor α (ERRα), thus controlling mitochondrial biogenesis and respiratory function [126,127]. In recent years, the investigation of the close connection existing between PGC-1α and estrogen-related receptor α (ERRα) has led to the development of molecules able to interfere with this pathway, thus exerting promising antitumor activity [126,128,129]. Among these, one of the most studied is XCT790, which acts as an ERRα inverse agonist. This compound has been demonstrated to drastically decrease mitochondrial biogenesis in cancer cells, reducing their proliferation and increasing their sensitivity to chemotherapeutic agents both in vitro and in vivo [130,131,132,133].

Recently, some inhibitors of c-Myc transcription factor have attracted considerable interest, although for many years it had been considered an “undruggable target”. This factor is able to integrate signals from different pathways, as well as modulating the expression of genes involved in metabolism, proliferation, apoptosis, and neoplastic transformation [134]. The inhibition of c-Myc function by small molecules can be achieved at different levels. Recently, inhibitors of BET bromodomain proteins able to inhibit c-Myc transcription have been developed. This class of inhibitors includes molecules such as JQ1 [135] and GSK525762, the clinical efficacy of which is currently under investigation in the treatment of solid tumors and hematopoietic malignancies (ClinicalTrials.gov: NCT01943851, NCT03266159). Another strategy to target c-Myc function is to pharmacologically inhibit its dimerization with Max protein, which is necessary for its activation. Small molecules able to inhibit c-Myc–Max complex formation include the compounds IIA6B17 [136], 10058-F4 [137], and omomyc, which can block the cell cycle and induce apoptosis in different tumor types, as well as reducing tumorigenesis in mouse models [138].

A high antitumor potential also characterizes the mTOR-related pathway inhibitors. Rapamycin (sirolimus), a natural product isolated from *Streptomyces hygroscopicus*, represents the first mTOR inhibitor to be identified, and it was initially FDA-approved for the prophylaxis of organ rejection in renal transplantation. Its limited solubility and consequent pharmacokinetic problems have reduced its clinical use. This has paved the way for the development of first-generation analogues of rapamycin, called rapalogs, such as temsirolimus and everolimus, which are both FDA-approved for the treatment of renal cell carcinoma (RCC). Second-generation analogues of rapamycin are defined as ATP-competitive mTOR kinase inhibitors, and they have been demonstrated to have greater selectivity in inhibiting mTOR catalytic activity compared to the previous class of compounds [121]. This new class of mTOR inhibitors includes molecules such as AZD8055 [139,140], INK-128 (sapanisertib) [141,142], OSI027 [143], and torin2 [144]. The latter is currently undergoing clinical studies in order to confirm its safety and efficacy in the treatment of advanced solid tumors and hematopietic malignancies.

Protein synthesis at the mitochondrial level has proven to be another promising target in cancer treatment. The mitochondrial translation process takes place at the level of mitoribosomes, which have been intensively investigated due to their peculiar structure, being different from that of cytoplasmic ribosomes or those anchored to the endoplasmic reticulum (ER). Interestingly, a close correlation has been observed between mitoribosomes and different pathologies such as cancer [145,146]. In this regard, several classes of antibiotics, such as aminoglycosides, macrolides, tetracyclines, lincosamides, and chloramphenicol, have been proven to be able to inhibit mitochondrial protein synthesis at the level of mitoribosomes. Such peculiar activity is based on the “endosymbiotic theory of mitochondrial evolution”, which hypothesizes that mitochondria evolved from engulfed aerobic bacteria [147]. Among the FDA-approved antibiotics that show the greatest potential in targeting mitochondrial cancer function are tigecycline and doxycycline. These molecules have been proven to reduce mitochondrial function and tumor stemness in many cancer models, both in vitro and in vivo [148,149,150,151,152,153,154]. In particular, in a clinical pilot study, doxycycline was shown to effectively reduce cancer stem cells in early breast cancer patients [155].

The effects of a recent class of antibiotics, oxazolidinones, from the progenitor linezolid, have been widely reported the scientific literature. These molecules have been found to potently inhibit mitochondrial protein synthesis [156], altering the fine homeostasis of this organelle, thus generating ROS and inducing apoptosis in cancer cells [157,158,159].

Recently, besides repurposing of FDA-approved antibiotics, the increase in knowledge about mitoribosomes’ structure and functioning has promoted the development of new molecules specifically designed to bind and inhibit cancer mitoribosomes, such as mitoriboscins [160]. 

In addition to protein synthesis, mitochondrial turnover is ensured by a dynamic balance between mitochondrial fission and fusion processes, of which the main mediators are GTPase dynamin-related protein 1 (DRP1) and Mitofusin-2 (MFN-2), respectively (Figure 5). A remarkable expression of DRP1, along with downregulation of MFN-2, is observed in cancer, resulting in an imbalance of mitochondrial dynamics towards fission and mitochondrial biogenesis [161]. The antitumor potential of targeting DRP1 has prompted oncologic research to identify small molecules able to modulate mitochondrial dynamics. The mitochondrial division inhibitor 1, Mdvi-1, a selective inhibitor of DRP1, has been demonstrated to be a powerful inhibitor of mitochondrial fission. This molecule has been proven to reduce oxidative metabolism and to arrest the cell cycle at the G2–M checkpoint, thus impairing cell proliferation, reducing stemness, and triggering apoptosis in breast [162], lung, and colon cancer cell lines [163] both in vitro and in vivo. Moreover, in a recent report, two ellipticine analogs, namely Drpitor1 and Drpitor1a, displayed a selective inhibitory activity against DRP1 by reducing mitochondrial fission, inhibiting proliferation of cancer cells, and inducing apoptotic death in both cancer cell cultures and xenograft mouse models [164].

## 3. Conclusions

Mitochondria play a pivotal role in cancer by influencing several steps in oncogenesis and tumor progression. Different mitochondrial metabolic pathways may adapt in order to regulate bioenergetic or anabolic functions, thus contributing to the metabolic rewiring of cancer cells. However, mitochondrial biology is complex and mitochondrial metabolism is very intricate and tightly regulated. Moreover, the structure of these organelles, delimited by a double membrane, limits the entry of molecules into the mitochondrial matrix, making the processes that take place in mitochondria quite difficult to target. A further challenge in the clinical development of mitochondrial inhibitors is the potential toxicity that nonspecific mitochondrial inhibition could trigger. Despite these issues, in recent decades, many researchers have aimed to develop molecules that act as safe mitochondrial metabolism inhibitors. In this review, we highlighted the promising antitumor potential of several molecules which have been proven to be effective in vitro and in vivo. Some of these compounds have displayed interesting results in preclinical or early clinical trials, others have been already approved by the FDA as anticancer agents, and for still others, which are FDA-approved for different therapeutic indications, their repurposing in the oncological field is being evaluated. Overall, this review points out the potential clinical usefulness of targeting mitochondrial metabolic pathways, and the reported promising evidence strongly encourages the deepening of knowledge on mitochondria, in order to provide more effective and better tolerated therapeutic alternatives than those currently applied in clinical practice.

## Abbreviations

ATP	Adenosine triphosphate
ADP	Adenosine diphosphate
NAD^+^	Nicotinamide adenine dinucleotide (oxidized form)
NADPH	Nicotinamide adenine dinucleotide (reduced form)
NADPH	Nicotinamide adenine dinucleotide phosphate (reduced form)
FDA	Food and Drug Administration
SLC25A1	solute carrier family 25 member 1 (citrate transporter)
SLC1A5	solute carrier family 1 member 5 (neutral amino acid transporter)
AML	acute myeloid leukemia
PI3K	Phosphoinositide 3-kinase
AKT	Protein kinase B

## References

[1] Warburg O. (1956). On the origin of cancer cells. Science.

[2] Seyfried T.N. (2015). Cancer as a mitochondrial metabolic disease. Front. Cell Dev. Biol..

[3] Lee E.Y., Muller W.J. (2010). Oncogenes and tumor suppressor genes. Cold Spring Harb. Perspect. Biol..

[4] Lu J., Sharma L.K., Bai Y.J. (2009). Implications of mitochondrial DNA mutations and mitochondrial dysfunction in tumorigenesis. Cell Res..

[5] Sciacovelli M., Frezza C. (2016). Oncometabolites: Unconventional triggers of oncogenic signalling cascades. Free Radic. Biol. Med..

[6] Collins R.R., Patel K., Putnam W.C., Kapur P., Rakheja D. (2017). Oncometabolites: A new paradigm for oncology, metabolism, and the clinical laboratory. Clin. Chem..

[7] Morin A., Letouzé E., Gimenez-Roqueplo A.P., Favier J. (2014). Oncometabolites-driven tumorigenesis: From genetics to targeted therapy. Int. J. Cancer.

[8] Catalina-Rodriguez O., Kolukula V.K., York Tomita A.P., Palmieri F., Wellstein A., Byers S., Giaccia A.J., Glasgow E., Albanese C., Avantaggiati M.L. (2012). The mitochondrial citrate transporter, CIC, is essential for mitochondrial homeostasis. Oncotarget.

[9] Hlouschek J., Hansel C., Jendrossek V., Matschke J. (2018). The Mitochondrial Citrate Carrier (SLC25A1) Sustains Redox Homeostasis and Mitochondrial Metabolism Supporting Radioresistance of Cancer Cells with Tolerance to Cycling Severe Hypoxia. Front. Oncol..

[10] Fernandez H.R., Gadre S.M., Tan M., Graham G.T., Mosaoa R., Ongkeko M.S., Kim K.A., Riggins R.B., Parasido E., Petrini I. (2018). The mitochondrial citrate carrier, SLC25A1, drives stemness and therapy resistance in non-small cell lung cancer. Cell Death Differ..

[11] Ochoa-Ruiz E., Diaz-Ruiz R. (2012). Anaplerosis in cancer: Another step beyond the warburg effect. Am. J. Mol. Boil..

[12] Song Z., Wei B., Lu C., Li P., Chen L. (2017). Glutaminase sustains cell survival via the regulation of glycolysis and glutaminolysis in colorectal cancer. Oncol. Lett..

[13] Avagliano A., Ruocco M.R., Aliotta F., Belviso I., Accurso A., Masone S., Montagnani S., Arcucci A. (2019). Mitochondrial Flexibility of Breast Cancers: A Growth Advantage and a Therapeutic Opportunity. Cells.

[14] Lane A.N., Higashi R.M., Fan T.W.-M. (2020). Metabolic reprogramming in tumors: Contributions of the tumor microenvironment. Genes Dis..

[15] Gouirand V., Guillaumond F., Vasseur S. (2018). Influence of the Tumor Microenvironment on Cancer Cells Metabolic Reprogramming. Front. Oncol..

[16] Lyssiotis C.A., Kimmelman A.C. (2017). Metabolic Interactions in the Tumor Microenvironment. Trends Cell Boil..

[17] Sotgia F., Whitaker-Menezes D., Martinez-Outschoorn U.E., Flomenberg N., Birbe R., Witkiewicz A.K., Howell A., Philp N.J., Pestell R.G., Lisanti M.P. (2012). Mitochondrial metabolism in cancer metastasis. Cell Cycle.

[18] Porporato P.E., Payen V.L., Pérez-Escuredo J., De Saedeleer C.J., Danhier P., Copetti T., Dhup S., Tardy M., Vazeille T., Bouzin C. (2014). A Mitochondrial Switch Promotes Tumor Metastasis. Cell Rep..

[19] Thomson T.M., Balcells C., Cascante M. (2019). Metabolic Plasticity and Epithelial-Mesenchymal Transition. J. Clin. Med..

[20] Bhattacharya D., Scimè A. (2019). Metabolic Regulation of Epithelial to Mesenchymal Transition: Implications for Endocrine Cancer. Front. Endocrinol..

[21] Yin S., Cheryan V.T., Xu L., Rishi A.K., Reddy K.B. (2017). Myc mediates cancer stem-like cells and EMT changes in triple negative breast cancers cells. PLoS ONE.

[22] Cho K.B., Cho M.K., Lee W.Y., Kang K.W. (2010). Overexpression of c-myc induces epithelial mesenchymal transition in mammary epithelial cells. Cancer Lett..

[23] Roshan M.K., Soltani A., Soleimani A., Kahkhaie K.R., Afshari A.R., Soukhtanloo M. (2019). Role of AKT and mTOR signaling pathways in the induction of epithelial-mesenchymal transition (EMT) process. Biochimie.

[24] Yoriki K., Mori T., Kokabu T., Matsushima H., Umemura S., Tarumi Y., Kitawaki J. (2019). Estrogen-related receptor alpha induces epithelial-mesenchymal transition through cancer-stromal interactions in endometrial cancer. Sci. Rep..

[25] Ashton T.M., McKenna W.G., Kunz-Schughart L.A., Higgins G.S. (2018). Oxidative Phosphorylation as an Emerging Target in Cancer Therapy. Clin. Cancer Res..

[26] Sohoni S., Ghosh P., Wang T., Kalainayakan S.P., Vidal C., Dey S., Konduri P.C., Zhang L. (2019). Elevated Heme Synthesis and Uptake Underpin Intensified Oxidative Metabolism and Tumorigenic Functions in Non–Small Cell Lung Cancer Cells. Cancer Res..

[27] Yadav N., Kumar S., Marlowe T., Chaudhary A., Kumar R., Wang J., O’malley J., Boland P., Jayanthi S., Kumar T.K.S. (2015). Oxidative phosphorylation-dependent regulation of cancer cell apoptosis in response to anticancer agents. Cell Death Dis..

[28] Dey R., Moraes C.T. (2000). Lack of Oxidative Phosphorylation and Low Mitochondrial Membrane Potential Decrease Susceptibility to Apoptosis and Do Not Modulate the Protective Effect of Bcl-xLin Osteosarcoma Cells. J. Boil. Chem..

[29] Fiorillo M., Tóth F., Brindisi M., Sotgia F., Lisanti M.P. (2020). Deferiprone (DFP) Targets Cancer Stem cell (CSC) Propagation by Inhibiting Mitochondrial Metabolism and Inducing ROS Production. Cells.

[30] Urra F.A., Córdova-Delgado M., Lapier M., Orellana-Manzano A., Acevedo-Arévalo L., Pessoa-Mahana H., González-Vivanco J.M., Martínez-Cifuentes M., Ramírez-Rodríguez O., Millas-Vargas J.P. (2016). Small structural changes on a hydroquinone scaffold determine the complex I inhibition or uncoupling of tumoral oxidative phosphorylation. Toxicol. Appl. Pharmacol..

[31] Degli Esposti M. (1998). Inhibitors of NADH–ubiquinone reductase: An overview. Biochim. Biophys. Acta (BBA) Bioenerg..

[32] Urra F.A., Muñoz F., Lovy A., Cárdenas C. (2017). The Mitochondrial Complex(I)ty of Cancer. Front. Oncol..

[33] Wheaton W.W., Weinberg S.E., Hamanaka R.B., Soberanes S., Sullivan L.B., Anso E., Glasauer A., Dufour E., Mutlu G.M., Budigner G.S. (2014). Metformin inhibits mitochondrial complex I of cancer cells to reduce tumorigenesis. eLife.

[34] Palorini R., Simonetto T., Cirulli C., Chiaradonna F. (2013). Mitochondrial Complex I Inhibitors and Forced Oxidative Phosphorylation Synergize in Inducing Cancer Cell Death. Int. J. Cell Boil..

[35] Schöckel L., Glasauer A., Basit F., Bitschar K., Truong H., Erdmann G., Algire C., Hägebarth A., Willems P.H., Kopitz C. (2015). Targeting mitochondrial complex I using BAY 87-2243 reduces melanoma tumor growth. Cancer Metab..

[36] Naguib A., Mathew G., Reczek C.R., Watrud K., Ambrico A., Herzka T., Salas I.C., Lee M.F., El-Amine N., Zheng W. (2018). Mitochondrial Complex I Inhibitors Expose a Vulnerability for Selective Killing of Pten-Null Cells. Cell Rep..

[37] Hadrava Vanova K., Kraus M., Neuzil J., Rohlena J. (2020). Mitochondrial complex II and reactive oxygen species in disease and therapy. Redox Rep..

[38] Kluckova K., Bezawork-Geleta A., Rohlena J., Dong L., Neuzil J. (2013). Mitochondrial complex II, a novel target for anti-cancer agents. Biochim. Biophys. Acta (BBA) Bioenerg..

[39] Gruber J., Staniek K., Krewenka C., Moldzio R., Patel A., Böhmdorfer S., Rosenau T., Gille L. (2014). Tocopheramine succinate and tocopheryl succinate: Mechanism of mitochondrial inhibition and superoxide radical production. Bioorg. Med. Chem..

[40] Min H.-Y., Jang H.-J., Park K.H., Hyun S.Y., Park S.J., Kim J.H., Son J., Kang S.S., Lee H.-Y. (2019). The natural compound gracillin exerts potent antitumor activity by targeting mitochondrial complex II. Cell Death Dis..

[41] Kawada M., Momose I., Someno T., Tsujiuchi G., Ikeda D. (2009). New atpenins, NBRI23477 A and B, inhibit the growth of human prostate cancer cells. J. Antibiot..

[42] Wang H., Huwaimel B., Verma K., Miller J., Germain T.M., Kinarivala N., Pappas D., Brookes P.S., Trippier P.C. (2017). Synthesis and Antineoplastic Evaluation of Mitochondrial Complex II (Succinate Dehydrogenase) Inhibitors Derived from Atpenin A5. ChemMedChem.

[43] Miyadera H., Shiomi K., Ui H., Yamaguchi Y., Masuma R., Tomoda H., Miyoshi H., Osanai A., Kita K., Ōmura S. (2003). Atpenins, potent and specific inhibitors of mitochondrial complex II (succinate-ubiquinone oxidoreductase). Proc. Natl. Acad. Sci. USA.

[44] Guo L., Shestov A.A., Worth A.J., Nath K., Nelson D.S., Leeper D.B., Glickson J.D., Blair I.A. (2015). Inhibition of Mitochondrial Complex II by the Anticancer Agent Lonidamine. J. Boil. Chem..

[45] Teicher B., Holden S., Herman T., Frei E. (1991). Modulation of alkylating agents by lonidamine in vivo. Semin. Oncol..

[46] Nath K., Nelson D.S., Heitjan D.F., Leeper D.B., Zhou R., Glickson J.D. (2015). Lonidamine induces intracellular tumor acidification and ATP depletion in breast, prostate and ovarian cancer xenografts and potentiates response to doxorubicin. NMR Biomed..

[47] Gupta N., Srivastava S.K. (2019). Atovaquone: An Antiprotozoal Drug Suppresses Primary and Resistant Breast Tumor Growth by Inhibiting HER2/β-Catenin Signaling. Mol. Cancer Ther..

[48] Gao X., Liu X., Shan W., Liu Q., Wang C., Zheng J., Yao H., Tang R., Zheng J. (2018). Anti-malarial atovaquone exhibits anti-tumor effects by inducing DNA damage in hepatocellular carcinoma. Am. J. Cancer Res..

[49] Fiorillo M., Lamb R., Tanowitz H.B., Mutti L., Krstic-Demonacos M., Cappello A.R., Martinez-Outschoorn U.E., Sotgia F., Lisanti M.P. (2016). Repurposing atovaquone: Targeting mitochondrial complex III and OXPHOS to eradicate cancer stem cells. Oncotarget.

[50] Paragliola R.M., Torino F., Papi G., Locantore P., Pontecorvi A., Corsello S.M. (2018). Role of Mitotane in Adrenocortical Carcinoma—Review and State of the art. Eur. Endocrinol..

[51] Hescot S., Slama A., Lombes A., Paci A., Remy H., Leboulleux S., Chadarevian R., Trabado S., Amazit L., Young J. (2013). Mitotane alters mitochondrial respiratory chain activity by inducing cytochrome c oxidase defect in human adrenocortical cells. Endocr. Relat. Cancer.

[52] Stevens J.J., Graham B., Dugo E., Berhaneselassie-Sumner B., Ndebele K., Tchounwou P.B. (2016). Arsenic Trioxide Induces Apoptosis via Specific Signaling Pathways in HT-29 Colon Cancer Cells. J. Cancer Sci. Ther..

[53] Sadaf N., Kumar N., Ali M., Ali V., Bimal S., Haque R., Bimal S. (2018). Arsenic trioxide induces apoptosis and inhibits the growth of human liver cancer cells. Life Sci..

[54] Diepart C., Karroum O., Magat J., Feron O., Verrax J., Calderon P.B., Grégoire V., Leveque P., Stockis J., Dauguet N. (2011). Arsenic Trioxide Treatment Decreases the Oxygen Consumption Rate of Tumor Cells and Radiosensitizes Solid Tumors. Cancer Res..

[55] Chang K.-J., Yang M.-H., Zheng J.-C., Li B., Nie W. (2016). Arsenic trioxide inhibits cancer stem-like cells via down-regulation of Gli1 in lung cancer. Am. J. Transl. Res..

[56] Esparza-Moltó P.B., Cuezva J.M. (2018). The Role of Mitochondrial H+-ATP Synthase in Cancer. Front. Oncol..

[57] Isidoro A., Martínez M., Fernández P.L., Ortega Á.D., Santamaría G., Chamorro M., Reed J.C., Cuezva J.M. (2004). Alteration of the bioenergetic phenotype of mitochondria is a hallmark of breast, gastric, lung and oesophageal cancer. Biochem. J..

[58] García-Ledo L., Nuevo-Tapioles C., Cuevas-Martín C., Martínez-Reyes I., Soldevilla B., González-Llorente L., Cuezva J.M. (2017). Overexpression of the ATPase Inhibitory Factor 1 Favors a Non-metastatic Phenotype in Breast Cancer. Front. Oncol..

[59] Sgarbi G., Barbato S., Costanzini A., Solaini G., Baracca A. (2018). The role of the ATPase inhibitor factor 1 (IF1) in cancer cells adaptation to hypoxia and anoxia. Biochim. Biophys. Acta (BBA) Bioenerg..

[60] Wang Y., Hou Q., Xiao G., Yang S., Di C., Si J., Zhou R., Ye Y., Zhang Y., Zhang H. (2017). Selective ATP hydrolysis inhibition in F1Fo ATP synthase enhances radiosensitivity in non-small-cell lung cancer cells (A549). Oncotarget.

[61] Fiorillo M., Lamb R., Tanowitz H.B., Cappello A.R., Martinez-Outschoorn U.E., Sotgia F., Lisanti M.P. (2016). Bedaquiline, an FDA-approved antibiotic, inhibits mitochondrial function and potently blocks the proliferative expansion of stem-like cancer cells (CSCs). Aging.

[62] Wu X., Li F., Wang X., Li C., Meng Q., Wang C., Huang J., Chen S., Zhu Z. (2018). Antibiotic bedaquiline effectively targets growth, survival and tumor angiogenesis of lung cancer through suppressing energy metabolism. Biochem. Biophys. Res. Commun..

[63] Gledhill J.R., Montgomery M.G., Leslie A.G., Walker J.E. (2007). Mechanism of inhibition of bovine F1-ATPase by resveratrol and related polyphenols. Proc. Natl. Acad. Sci. USA.

[64] Huang T.-C., Chang H.-Y., Hsu C.-H., Kuo W.-H., Chang K.-J., Juan H.-F. (2008). Targeting Therapy for Breast Carcinoma by ATP Synthase Inhibitor Aurovertin B. J. Proteome Res..

[65] Frattaruolo L., Fiorillo M., Brindisi M., Curcio R., Dolce V., Lacret R., Truman A.W., Sotgia F., Lisanti M.P., Cappello A.R. (2019). Thioalbamide, A Thioamidated Peptide from Amycolatopsis alba, Affects Tumor Growth and Stemness by Inducing Metabolic Dysfunction and Oxidative Stress. Cells.

[66] Dahlem C., Siow W.X., Lopatniuk M., Tse W.K., Kessler S.M., Kirsch S.H., Hoppstädter J., Vollmar A.M., Müller R., Luzhetskyy A. (2020). Thioholgamide A, a New Anti-Proliferative Anti-Tumor Agent, Modulates Macrophage Polarization and Metabolism. Cancers.

[67] Takase S., Kurokawa R., Kondoh Y., Honda K., Suzuki T., Kawahara T., Ikeda H., Dohmae N., Osada H., Shin-Ya K. (2019). Mechanism of Action of Prethioviridamide, an Anticancer Ribosomally Synthesized and Post-Translationally Modified Peptide with a Polythioamide Structure. ACS Chem. Boil..

[68] Turdo A., Porcelli G., D’Accardo C., Franco S.D., Verona F., Forte S., Giuffrida D., Memeo L., Todaro M., Stassi G. (2020). Metabolic Escape Routes of Cancer Stem Cells and Therapeutic Opportunities. Cancers.

[69] Hirpara J., Eu J.Q., Tan J.K.M., Wong A.L., Clement M.-V., Kong L.R., Ohi N., Tsunoda T., Qu J., Goh B.C. (2019). Metabolic reprogramming of oncogene-addicted cancer cells to OXPHOS as a mechanism of drug resistance. Redox Boil..

[70] Lee M., Hirpara J.L., Eu J.-Q., Sethi G., Wang L., Goh B.-C., Wong A.L.-A. (2019). Targeting STAT3 and oxidative phosphorylation in oncogene-addicted tumors. Redox Boil..

[71] Lee H.-J., Zhuang G., Cao Y., Du P., Kim H.-J., Settleman J. (2014). Drug Resistance via Feedback Activation of Stat3 in Oncogene-Addicted Cancer Cells. Cancer Cell.

[72] Wong A.L., Hirpara J.L., Pervaiz S., Eu J.-Q., Sethi G., Goh B.C. (2017). Do STAT3 inhibitors have potential in the future for cancer therapy?. Expert Opin. Investig. Drugs.

[73] Genini D., Brambilla L., Laurini E., Merulla J., Civenni G., Pandit S., D’Antuono R., Perez L., Levy D.E., Pricl S. (2017). Mitochondrial dysfunction induced by a SH2 domain-targeting STAT3 inhibitor leads to metabolic synthetic lethality in cancer cells. Proc. Natl. Acad. Sci. USA.

[74] Qin J.-J., Yan L., Zhang J., Zhang W.-D. (2019). STAT3 as a potential therapeutic target in triple negative breast cancer: A systematic review. J. Exp. Clin. Cancer Res..

[75] Wong A., Soo R.A., Tan D., Lee S.C., Lim J., Marban P., Kong L.R., Lee Y., Wang L., Thuya W.L. (2015). Phase I and biomarker study of OPB-51602, a novel signal transducer and activator of transcription (STAT) 3 inhibitor, in patients with refractory solid malignancies. Ann. Oncol..

[76] Swenson S.A., Moore C.M., Marcero J.R., Medlock A.E., Reddi A.R., Khalimonchuk O. (2020). From Synthesis to Utilization: The Ins and Outs of Mitochondrial Heme. Cells.

[77] Azuma M., Kabe Y., Kuramori C., Kondo M., Yamaguchi Y., Handa H. (2008). Adenine nucleotide translocator transports haem precursors into mitochondria. PLoS ONE.

[78] Hooda J., Cadinu D., Alam M.M., Shah A., Cao T.M., Sullivan L.A., Brekken R., Zhang L. (2013). Enhanced heme function and mitochondrial respiration promote the progression of lung cancer cells. PLoS ONE.

[79] Kalainayakan S.P., FitzGerald K.E., Konduri P.C., Vidal C., Zhang L. (2018). Essential roles of mitochondrial and heme function in lung cancer bioenergetics and tumorigenesis. Cell Biosci..

[80] Ghosh P., Vidal C., Dey S., Zhang L. (2020). Mitochondria Targeting as an Effective Strategy for Cancer Therapy. Int. J. Mol. Sci..

[81] Lee P.J., Woo S.J., Yoo H.M., Cho N., Kim H.P. (2019). Differential Mechanism of ATP Production Occurs in Response to Succinylacetone in Colon Cancer Cells. Molecules.

[82] Weinbach E.C., Ebert P.S. (1985). Effects of succinylacetone on growth and respiration of L1210 leukemia cells. Cancer Lett..

[83] Ebert P.S., Hess R.A., Frykholm B.C., Tschudy D.P. (1979). Succinylacetone, a potent inhibitor of heme biosynthesis: Effect on cell growth, heme content and δ-aminolevulinic acid dehydratase activity of malignant murine erythroleukemia cells. Biochem. Biophys. Res. Commun..

[84] Ye W., Zhang L. (2004). Heme controls the expression of cell cycle regulators and cell growth in HeLa cells. Biochem. Biophys. Res. Commun..

[85] Kalainayakan S.P., Ghosh P., Dey S., Fitzgerald K.E., Sohoni S., Konduri P.C., Garrossian M., Liu L., Zhang L. (2019). Cyclopamine tartrate, a modulator of hedgehog signaling and mitochondrial respiration, effectively arrests lung tumor growth and progression. Sci. Rep..

[86] Urban D.J., Martinez N.J., Davis M.I., Brimacombe K.R., Cheff D.M., Lee T.D., Henderson M.J., Titus S.A., Pragani R., Rohde J.M. (2017). Assessing inhibitors of mutant isocitrate dehydrogenase using a suite of pre-clinical discovery assays. Sci. Rep..

[87] Fujii T., Khawaja M.R., DiNardo C.D., Atkins J.T., Janku F. (2016). Targeting isocitrate dehydrogenase (IDH) in cancer. Discov. Med..

[88] Chen J., Yang J., Cao P. (2016). The Evolving Landscape in the Development of Isocitrate Dehydrogenase Mutant Inhibitors. Mini Rev. Med. Chem..

[89] Cho Y.S., Levell J.R., Liu G., Caferro T., Sutton J., Shafer C.M., Costales A., Manning J.R., Zhao Q., Sendzik M. (2017). Discovery and Evaluation of Clinical Candidate IDH305, a Brain Penetrant Mutant IDH1 Inhibitor. ACS Med. Chem. Lett..

[90] Dao10 K.-H., Kantarjian11 H.M., Kelly12 P., Sweeney12 J., Watson12 C., Mohamed12 H., Cortes11 J.E. (2018). Phase 1 study of the IDH1m inhibitor FT-2102 as a single agent in patients with IDH1m acute myeloid leukemia (AML) or myelodysplastic syndrome (MDS). Headache.

[91] Chaturvedi A., Gupta C., Gabdoulline R., Borchert N.M., Goparaju R., Kaulfuss S., Görlich K., Schottmann R., Othman B., Welzenbach J. (2020). Synergistic activity of IDH1 inhibitor BAY1436032 with azacitidine in IDH1 mutant acute myeloid leukemia. Haematologica.

[92] Chaturvedi A., Herbst L., Pusch S., Klett L., Goparaju R., Stichel D., Kaulfuss S., Panknin O., Zimmermann K., Toschi L. (2017). Pan-mutant-IDH1 inhibitor BAY1436032 is highly effective against human IDH1 mutant acute myeloid leukemia in vivo. Leukemia.

[93] Wise D.R., DeBerardinis R.J., Mancuso A., Sayed N., Zhang X.-Y., Pfeiffer H.K., Nissim I., Daikhin E., Yudkoff M., McMahon S.B. (2008). Myc regulates a transcriptional program that stimulates mitochondrial glutaminolysis and leads to glutamine addiction. Proc. Natl. Acad. Sci. USA.

[94] Wang R., Dillon C.P., Shi L.Z., Milasta S., Carter R., Finkelstein D., McCormick L.L., Fitzgerald P., Chi H., Munger J. (2011). The transcription factor Myc controls metabolic reprogramming upon T lymphocyte activation. Immunity.

[95] Mohamed A., Deng X., Khuri F.R., Owonikoko T.K., Mohammed M.A. (2014). Altered Glutamine Metabolism and Therapeutic Opportunities for Lung Cancer. Clin. Lung Cancer.

[96] Márquez J., Alonso F.J., Matés J.M., Segura J.A., Martín-Rufián M., Campos-Sandoval J.A., Márquez J.D. (2017). Glutamine Addiction In Gliomas. Neurochem. Res..

[97] Scalise M., Pochini L., Galluccio M., Console L., Indiveri C. (2017). Glutamine Transport and Mitochondrial Metabolism in Cancer Cell Growth. Front. Oncol..

[98] Lu J., Chen M., Tao Z., Gao S., Li Y., Cao Y., Lu C., Zou X. (2017). Effects of targeting SLC1A5 on inhibiting gastric cancer growth and tumor development in vitro and in vivo. Oncotarget.

[99] Wang Q., Beaumont K.A., Otte N.J., Font J., Bailey C.G., van Geldermalsen M., Sharp D.M., Tiffen J.C., Ryan R.M., Jormakka M. (2014). Targeting glutamine transport to suppress melanoma cell growth. Int. J. Cancer.

[100] Hassanein M., Qian J., Hoeksema M.D., Wang J., Jacobovitz M., Ji X., Harris F.T., Harris B.K., Boyd K.L., Chen H. (2015). Targeting SLC1a5-mediated glutamine dependence in non-small cell lung cancer. Int. J. Cancer.

[101] Hassanein M., Hoeksema M.D., Shiota M., Qian J., Harris B.K., Chen H., Clark J.E., Alborn W.E., Eisenberg R., Massion P.P. (2012). SLC1A5 mediates glutamine transport required for lung cancer cell growth and survival. Clin. Cancer Res..

[102] Lin J., Yang T., Peng Z., Xiao H., Jiang N., Zhang L., Dickerson C., Wu P., Pan Q. (2018). SLC1A5 Silencing Inhibits Esophageal Cancer Growth via Cell Cycle Arrest and Apoptosis. Cell. Physiol. Biochem..

[103] Yoo H.C., Park S.J., Nam M., Kang J., Kim K., Yeo J.H., Kim J.-K., Heo Y., Lee H.S., Lee M.Y. (2020). A Variant of SLC1A5 Is a Mitochondrial Glutamine Transporter for Metabolic Reprogramming in Cancer Cells. Cell Metab..

[104] Willems L., Jacque N., Jacquel A., Neveux N., Trovati Maciel T., Lambert M., Schmitt A., Poulain L., Green A.S., Uzunov M. (2013). Inhibiting glutamine uptake represents an attractive new strategy for treating acute myeloid leukemia. Blood.

[105] Nicklin P., Bergman P., Zhang B., Triantafellow E., Wang H., Nyfeler B., Yang H., Hild M., Kung C., Wilson C.J. (2009). Bidirectional transport of amino acids regulates mTOR and autophagy. Cell.

[106] Schulte M.L., Fu A., Zhao P., Li J., Geng L., Smith S.T., Kondo J., Coffey R.J., Johnson M.O., Rathmell J.C. (2018). Pharmacological blockade of ASCT2-dependent glutamine transport leads to antitumor efficacy in preclinical models. Nat. Med..

[107] Song M., Kim S.-H., Im C.Y., Hwang H.-J. (2018). Recent Development of Small Molecule Glutaminase Inhibitors. Curr. Top. Med. Chem..

[108] Xu X., Meng Y., Li L., Xu P., Wang J., Li Z., Bian J. (2018). Overview of the Development of Glutaminase Inhibitors: Achievements and Future Directions. J. Med. Chem..

[109] Matés J.M., Di Paola F.J., Campos-Sandoval J.A., Mazurek S., Márquez J. (2020). Therapeutic targeting of glutaminolysis as an essential strategy to combat cancer. Seminars in Cell & Developmental Biology.

[110] Guo L., Zhou B., Liu Z., Xu Y., Lu H., Xia M., Guo E., Shan W., Chen G., Wang C. (2016). Blockage of glutaminolysis enhances the sensitivity of ovarian cancer cells to PI3K/mTOR inhibition involvement of STAT3 signaling. Tumor Boil..

[111] Koch K., Hartmann R., Tsiampali J., Uhlmann C., Nickel A.-C., He X., Kamp M.A., Sabel M., Barker R.A., Steiger H.J. (2020). A comparative pharmaco-metabolomic study of glutaminase inhibitors in glioma stem-like cells confirms biological effectiveness but reveals differences in target-specificity. Cell Death Discov..

[112] Boysen G., Jamshidi-Parsian A., Davis M.A., Siegel E.R., Simecka C.M., Kore R.A., Dings R.P., Griffin R.J. (2019). Glutaminase inhibitor CB-839 increases radiation sensitivity of lung tumor cells and human lung tumor xenografts in mice. Int. J. Radiat. Boil..

[113] Zacharias N.M., McCullough C., Shanmugavelandy S., Lee J., Lee Y., Dutta P., McHenry J., Nguyen L., Norton W., Jones L.W. (2017). Metabolic Differences in Glutamine Utilization Lead to Metabolic Vulnerabilities in Prostate Cancer. Sci. Rep..

[114] Gross M.I., Demo S.D., Dennison J.B., Chen L., Chernov-Rogan T., Goyal B., Janes J.R., Laidig G.J., Lewis E.R., Li J. (2014). Antitumor activity of the glutaminase inhibitor CB-839 in triple-negative breast cancer. Mol. Cancer Ther..

[115] Lackner L.L., Nunnari J. (2010). Small molecule inhibitors of mitochondrial division: Tools that translate basic biological research into medicine. Chem. Boil..

[116] Tan Z., Luo X., Xiao L., Tang M., Bode A.M., Dong Z., Cao Y. (2016). The role of PGC1α in cancer metabolism and its therapeutic implications. Mol. Cancer Ther..

[117] Zong W.-X., Rabinowitz J.D., White E. (2016). Mitochondria and cancer. Mol. Cell.

[118] Li F., Wang Y., Zeller K.I., Potter J.J., Wonsey D.R., O’Donnell K.A., Kim J.-W., Yustein J.T., Lee L.A., Dang C.V. (2005). Myc stimulates nuclearly encoded mitochondrial genes and mitochondrial biogenesis. Mol. Cell. Boil..

[119] LeBleu V.S., O’Connell J.T., Herrera K.N.G., Wikman H., Pantel K., Haigis M.C., De Carvalho F.M., Damascena A., Chinen L.T.D., Rocha R.M. (2014). PGC-1α mediates mitochondrial biogenesis and oxidative phosphorylation in cancer cells to promote metastasis. Nat. Cell Biol..

[120] De Luca A., Fiorillo M., Peiris-Pagès M., Ozsvari B., Smith D.L., Sanchez-Alvarez R., Martinez-Outschoorn U.E., Cappello A.R., Pezzi V., Lisanti M.P. (2015). Mitochondrial biogenesis is required for the anchorage-independent survival and propagation of stem-like cancer cells. Oncotarget.

[121] Zaytseva Y.Y., Valentino J.D., Gulhati P., Evers B.M. (2012). mTOR inhibitors in cancer therapy. Cancer Lett..

[122] Laplante M., Sabatini D.M. (2009). mTOR signaling at a glance. J. Cell Sci..

[123] Laplante M., Sabatini D.M. (2012). mTOR signaling in growth control and disease. Cell.

[124] Morita M., Gravel S.-P., Hulea L., Larsson O., Pollak M., St-Pierre J., Topisirovic I. (2015). mTOR coordinates protein synthesis, mitochondrial activity and proliferation. Cell Cycle.

[125] Luo C., Widlund H.R., Puigserver P. (2016). PGC-1 coactivators: Shepherding the mitochondrial biogenesis of tumors. Trends Cancer.

[126] Scarpulla R.C. (2011). Metabolic control of mitochondrial biogenesis through the PGC-1 family regulatory network. Biochim. Biophys. Acta (BBA) Bioenerg..

[127] Yun C.W., Han Y.-S., Lee S.H. (2019). PGC-1α controls mitochondrial biogenesis in drug-resistant colorectal cancer cells by regulating endoplasmic reticulum stress. Int. J. Mol. Sci..

[128] Lynch C., Zhao J., Sakamuru S., Zhang L., Huang R., Witt K.L., Merrick B.A., Teng C.T., Xia M. (2019). Identification of compounds that inhibit estrogen-related receptor alpha signaling using high-throughput screening assays. Molecules.

[129] Brindisi M., Fiorillo M., Frattaruolo L., Sotgia F., Lisanti M.P., Cappello A.R. (2020). Cholesterol and Mevalonate: Two Metabolites Involved in Breast Cancer Progression and Drug Resistance through the ERRα Pathway. Cells.

[130] Wu Y.-M., Chen Z.-J., Jiang G.-M., Zhang K.-S., Liu Q., Liang S.-W., Zhou Y., Huang H.-B., Du J., Wang H. (2016). Inverse agonist of estrogen-related receptor α suppresses the growth of triple negative breast cancer cells through ROS generation and interaction with multiple cell signaling pathways. Oncotarget.

[131] Casaburi I., Avena P., De Luca A., Chimento A., Sirianni R., Malivindi R., Rago V., Fiorillo M., Domanico F., Campana C. (2015). Estrogen related receptor α (ERRα) a promising target for the therapy of adrenocortical carcinoma (ACC). Oncotarget.

[132] Wu F., Wang J., Wang Y., Kwok T.-T., Kong S.-K., Wong C. (2009). Estrogen-related receptor α (ERRα) inverse agonist XCT-790 induces cell death in chemotherapeutic resistant cancer cells. Chem. Interact..

[133] Kokabu T., Mori T., Matsushima H., Yoriki K., Kataoka H., Tarumi Y., Kitawaki J. (2019). Antitumor effect of XCT790, an ERRα inverse agonist, on ERα-negative endometrial cancer cells. Cell. Oncol..

[134] Carabet L.A., Rennie P.S., Cherkasov A. (2019). Therapeutic inhibition of Myc in cancer. Structural bases and computer-aided drug discovery approaches. Int. J. Mol. Sci..

[135] da Motta L.L., Ledaki I., Purshouse K., Haider S., De Bastiani M.A., Baban D., Morotti M., Steers G., Wigfield S., Bridges E. (2016). The BET inhibitor JQ1 selectively impairs tumour response to hypoxia and downregulates CA9 and angiogenesis in triple negative breast cancer. Oncogene.

[136] Lu X., Vogt P.K., Boger D.L., Lunec J. (2008). Disruption of the MYC transcriptional function by a small-molecule antagonist of MYC/MAX dimerization. Oncol. Rep..

[137] Huang M.-J., Cheng Y.-c., Liu C.-R., Lin S., Liu H.E. (2006). A small-molecule c-Myc inhibitor, 10058-F4, induces cell-cycle arrest, apoptosis, and myeloid differentiation of human acute myeloid leukemia. Exp. Hematol..

[138] Demma M.J., Mapelli C., Sun A., Bodea S., Ruprecht B., Javaid S., Wiswell D., Muise E., Chen S., Zelina J. (2019). Omomyc reveals new mechanisms to inhibit the MYC oncogene. Mol. Cell. Boil..

[139] Kauffman E.C., Lang M., Rais-Bahrami S., Gupta G.N., Wei D., Yang Y., Sourbier C., Srinivasan R. (2019). Preclinical efficacy of dual mTORC1/2 inhibitor AZD8055 in renal cell carcinoma harboring a TFE3 gene fusion. BCM Cancer.

[140] Chresta C.M., Davies B.R., Hickson I., Harding T., Cosulich S., Critchlow S.E., Vincent J.P., Ellston R., Jones D., Sini P. (2010). AZD8055 is a potent, selective, and orally bioavailable ATP-competitive mammalian target of rapamycin kinase inhibitor with in vitro and in vivo antitumor activity. Cancer Res..

[141] Li C., Cui J.-F., Chen M.-B., Liu C.-Y., Liu F., Zhang Q.-D., Zou J., Lu P.-H. (2015). The preclinical evaluation of the dual mTORC1/2 inhibitor INK-128 as a potential anti-colorectal cancer agent. Cancer Biol. Ther..

[142] Zhang H., Dou J., Yu Y., Zhao Y., Fan Y., Cheng J., Xu X., Liu W., Guan S., Chen Z. (2015). mTOR ATP-competitive inhibitor INK128 inhibits neuroblastoma growth via blocking mTORC signaling. Apoptosis.

[143] Bhagwat S.V., Gokhale P.C., Crew A.P., Cooke A., Yao Y., Mantis C., Kahler J., Workman J., Bittner M., Dudkin L. (2011). Preclinical characterization of OSI-027, a potent and selective inhibitor of mTORC1 and mTORC2: Distinct from rapamycin. Mol. Cancer Ther..

[144] Liu Q., Xu C., Kirubakaran S., Zhang X., Hur W., Liu Y., Kwiatkowski N.P., Wang J., Westover K.D., Gao P. (2013). Characterization of Torin2, an ATP-competitive inhibitor of mTOR, ATM, and ATR. Cancer Res..

[145] De Silva D., Tu Y.-T., Amunts A., Fontanesi F., Barrientos A. (2015). Mitochondrial ribosome assembly in health and disease. Cell Cycle.

[146] Martin T.D., Cook D.R., Choi M.Y., Li M.Z., Haigis K.M., Elledge S.J. (2017). A role for mitochondrial translation in promotion of viability in K-Ras mutant cells. Cell Rep..

[147] Roger A.J., Muñoz-Gómez S.A., Kamikawa R. (2017). The origin and diversification of mitochondria. Curr. Boil..

[148] Dijk S.N., Protasoni M., Elpidorou M., Kroon A.M., Taanman J.-W. (2020). Mitochondria as target to inhibit proliferation and induce apoptosis of cancer cells: The effects of doxycycline and gemcitabine. Sci. Rep..

[149] Son K., Fujioka S., Iida T., Furukawa K., Fujita T., Yamada H., Chiao P.J., Yanaga K. (2009). Doxycycline induces apoptosis in PANC-1 pancreatic cancer cells. Anticancer. Res..

[150] Lin C.C., Lo M.C., Moody R.R., Stevers N.O., Tinsley S.L., Sun D. (2018). Doxycycline targets aldehyde dehydrogenase-positive breast cancer stem cells. Oncol. Rep..

[151] Dong Z., Abbas M.N., Kausar S., Yang J., Li L., Tan L., Cui H. (2019). Biological functions and molecular mechanisms of antibiotic tigecycline in the treatment of cancers. Int. J. Mol. Sci..

[152] Arora R., Jain S., Rahimi H. (2019). Evaluating the efficacy of Tigecycline to target multiple cancer-types: A Review. STEM Fellowsh. J..

[153] Lu Z., Xu N., He B., Pan C., Lan Y., Zhou H., Liu X. (2017). Inhibition of autophagy enhances the selective anti-cancer activity of tigecycline to overcome drug resistance in the treatment of chronic myeloid leukemia. J. Exp. Clin. Cancer Res..

[154] Xiong Y., Liu W., Huang Q., Wang J., Wang Y., Li H., Fu X. (2018). Tigecycline as a dual inhibitor of retinoblastoma and angiogenesis via inducing mitochondrial dysfunctions and oxidative damage. Sci. Rep..

[155] Scatena C., Roncella M., Di Paolo A., Aretini P., Menicagli M., Fanelli G., Marini C., Mazzanti C.M., Ghilli M., Sotgia F. (2018). Doxycycline, an inhibitor of mitochondrial biogenesis, effectively reduces cancer stem cells (CSCs) in early breast cancer patients: A clinical pilot study. Front. Oncol..

[156] McKee E., Ferguson M., Bentley A., Marks T.A. (2006). Inhibition of mammalian mitochondrial protein synthesis by oxazolidinones. Antimicrob. Agents Chemother..

[157] Nagiec E.E., Wu L., Swaney S.M., Chosay J.G., Ross D.E., Brieland J.K., Leach K.L. (2005). Oxazolidinones inhibit cellular proliferation via inhibition of mitochondrial protein synthesis. Antimicrob. Agents Chemother..

[158] Hedaya O.M., Mathew P.M., Mohamed F.H., Phillips O.A., Luqmani Y.A. (2016). Antiproliferative activity of a series of 5-(1H-1, 2, 3-triazolyl) methyl-and 5-acetamidomethyl-oxazolidinone derivatives. Mol. Med. Rep..

[159] Armentano B., Curcio R., Brindisi M., Mancuso R., Rago V., Ziccarelli I., Frattaruolo L., Fiorillo M., Dolce V., Gabriele B. (2020). 5-(Carbamoylmethylene)-oxazolidin-2-ones as a promising class of heterocycles inducing apoptosis triggered by increased ros levels and mitochondrial dysfunction in breast and cervical cancer. Biomedicines.

[160] Ozsvari B., Fiorillo M., Bonuccelli G., Cappello A.R., Frattaruolo L., Sotgia F., Trowbridge R., Foster R., Lisanti M.P. (2017). Mitoriboscins: Mitochondrial-based therapeutics targeting cancer stem cells (CSCs), bacteria and pathogenic yeast. Oncotarget.

[161] Grandemange S., Herzig S., Martinou J.-C. (2009). Mitochondrial dynamics and cancer. Seminars in Cancer Biology.

[162] Peiris-Pagès M., Bonuccelli G., Sotgia F., Lisanti M.P. (2018). Mitochondrial fission as a driver of stemness in tumor cells: mDIVI1 inhibits mitochondrial function, cell migration and cancer stem cell (CSC) signalling. Oncotarget.

[163] Dai W., Wang G., Chwa J., Oh M.E., Abeywardana T., Yang Y., Wang Q.A., Jiang L. (2020). Mitochondrial division inhibitor (mdivi-1) decreases oxidative metabolism in cancer. Br. J. Cancer.

[164] Wu D., Dasgupta A., Chen K.H., Neuber-Hess M., Patel J., Hurst T.E., Mewburn J.D., Lima P.D., Alizadeh E., Martin A. (2020). Identification of novel dynamin-related protein 1 (Drp1) GTPase inhibitors: Therapeutic potential of Drpitor1 and Drpitor1a in cancer and cardiac ischemia-reperfusion injury. FASEB J..

